# Sliding into Place: The Lymphatic Vessel Endothelial Hyaluronan Receptor LYVE-1 and Its Role as a Mediator of Cell Entry and Trafficking in the Lymphatics

**DOI:** 10.3390/biom16060782

**Published:** 2026-05-26

**Authors:** David G. Jackson

**Affiliations:** MRC Translational Immune Discovery Unit, MRC Weatherall Institute of Molecular Medicine, University of Oxford, Oxford OX3 9DS, UK; david.jackson@imm.ox.ac.uk

**Keywords:** LYVE-1, lymphatic endothelium, hyaluronan, receptor, immune cell, dendritic cell, trafficking, binding

## Abstract

Hyaluronan (HA) receptors are expressed in a wide variety of different tissues and have long been known to support the critical cellular functions of adhesion and motility, in addition to a range of different physiological and pathological processes, including immunity, inflammation and tumour metastasis. In recent years, LYVE-1, an HA receptor largely but not exclusively restricted to the endothelia of lymphatic capillaries, has been shown to mediate the entry of immune cells through lymphatic endothelial junctions by engaging with their surface HA glycocalyx, itself anchored to the immune cell membrane by the closely related receptor CD44. Although similar to CD44 in primary sequence, LYVE-1 is functionally distinct, with a mutually exclusive pattern of tissue expression and a marked dependence on avidity for engagement with the long chains of HA—achieved primarily through receptor clustering. Here, we review key data that have defined the in vitro and in vivo functions of LYVE-1, including recent high-resolution crystal structures that have revealed its unusual and reversible “sliding” mode of interaction with HA, as distinct from the conventional “sticking” interaction in CD44. Lastly, we consider the emerging functions of LYVE-1 in sites beyond the lymphatics, namely tissue-resident macrophages and the specialised blood vessels of certain organs, and its potential as a therapeutic target.

## 1. Introduction

Interactions between the glycosaminoglycan hyaluronan (HA) and cell surface receptors of the HA-binding Link superfamily underlie fundamental processes of cell: cell and cell–matrix adhesion throughout the course of development, regeneration, inflammation and immunity [1,2,3]. In doing so, they tether immotile tissue resident cells such as fibroblasts and epithelia to the large insoluble scaffolds of HA and its Link superfamily binding partners Aggrecan, Versican, and the HAPLNs within interstitial and basement membrane matrices [4]. In the blood vasculature, the well-known HA receptor CD44 helps orchestrate the extravasation of circulating T cells and neutrophils within inflamed tissues by docking with deposits of the glycosaminoglycan sequestered on the endothelial surface of postcapillary venules [5]. Furthermore, CD44 anchors a polyanionic HA coat (glycocalyx) on the surface of motile immune cells, including dendritic cells (DCs) and monocyte/macrophages, which facilitates their amoeboid-like migration through tissue parenchyma through mutual charge repulsion [6]. Indeed, a potential role in HA-mediated cell adhesion has also been reported for the Link superfamily receptor HARE, AKA Stabilin-2, and FEL-2 in vascular endothelium. However, the primary function of HARE appears to be the endocytosis of HA for terminal degradation in the liver, spleen and lymph nodes, following its initial uptake and turnover via CD44 in peripheral tissue [7,8,9,10].

More recently, LYVE-1 has been recognised as a third Link superfamily HA receptor, with a pattern of expression distinct from that of CD44, and a primary localisation within the lymphatic vasculature [11]. Whereas CD44 mediates extravasation of immune cells in the blood vasculature, LYVE-1 mediates their intravasation in the lymphatics, most notably in the case of DCs, which migrate from the tissues to draining lymph nodes for the generation of protective immune responses [12,13,14,15,16]. Given that lymphatics are also a key route for the uptake of HA following its turnover in tissues and transport for terminal degradation in lymph nodes, spleen, liver and kidney via HARE endocytosis [8,17,18,19,20], LYVE-1 may well play some role in these processes (see ref. [21] for discussion). Besides lymphatics, LYVE-1 is expressed in tissue resident macrophages [22] and specialised blood vessels in the liver (i.e., sinusoids) and lung [23,24,25], where its particular functions remain to be fully elucidated. For the purposes of brevity, however, these latter aspects are covered only briefly here. Rather, this review focusses mainly on the role of LYVE-1 in lymphatics, beginning with the discovery of the receptor and the avidity-dependent nature of LYVE-1 • HA binding, followed by its in vivo functions in lymphatic trafficking and the repair of inflammatory injury, and ending with new insights into the precise molecular details of LYVE-1 • HA interactions revealed by recent structural and biophysical analyses that help explain its unusual mechanism of action [26].

## 2. Discovery of LYVE-1 as a Lymphatic Endothelial HA Receptor

**Figure 1:**
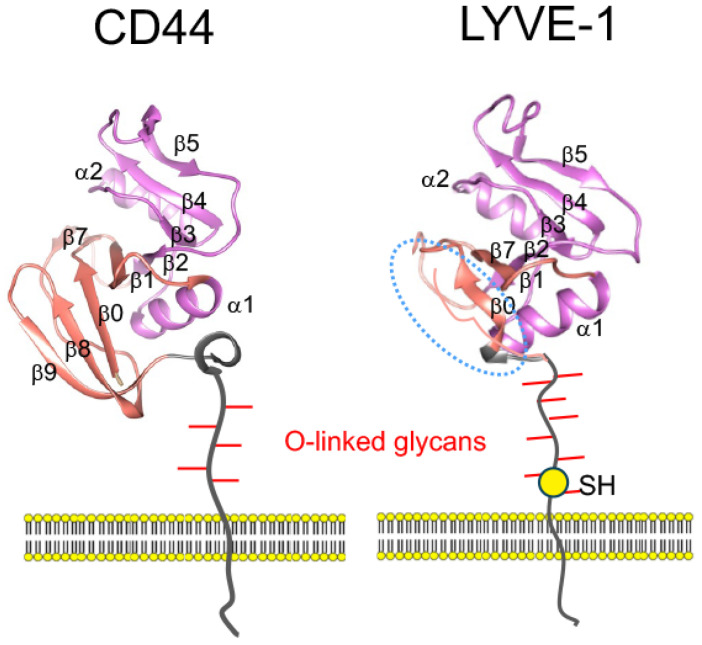
The 3D structures of hLYVE-1 and its closest homologue hCD44, with the extended HA-binding Link domain in each case shown in ribbon diagram format. The consensus Link module is coloured pink and the N- and C-terminal extensions coloured salmon. The blue stippling highlights the absence of β8 and β9 strands in LYVE-1 (see text). The stalk-like membrane proximal domains, which are unstructured and decorated with O-linked glycans (red lines), are shown in black. Unlike CD44, LYVE-1 carries an unpaired cysteine residue (yellow circle), through which it forms homodimers via intermolecular disulphide bonding (SH corresponds to the free sulfhydryl group). Figure is adapted from ref. [16] with permission from the publishers.

It was in the context of vascular trafficking that LYVE-1 was originally discovered, as part of an initiative to identify new candidate HA-binding proteins sharing sequence homology with CD44 and other Link superfamily receptors by probing the EST databases [11]. When assembled from appropriate overlapping ESTs, the full-length *Lyve1* transcript encoded a 340-residue receptor with an overall sequence similarity to CD44 of approximately 44%. This comprised an N-terminal HA-binding Link domain with the characteristic alpha/beta fold of six beta strands (β1–6) and two alpha chains (α1 and α2) stabilised by two conserved disulphides, followed by a serine-/threonine-rich membrane-proximal domain and a 63-residue cytoplasmic tail [11] (Figure 1).

**Figure 2:**
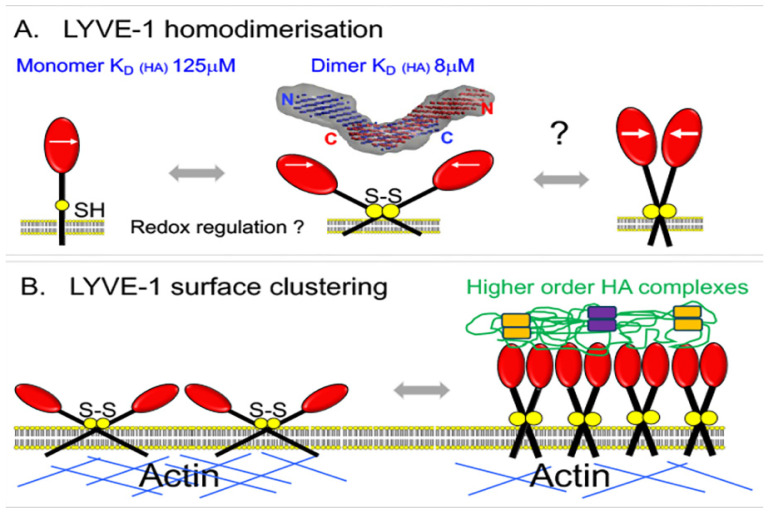
Key features of LYVE-1 that regulate its avidity-dependent interaction with HA in lymphatic endothelium. (**A**) Formation of disulphide-linked homodimers via the free cysteine residue in the membrane-proximal domain that is critical for HA-binding in vivo, and which increases HA-binding affinity by approx. 16-fold as estimated from surface plasmon resonance measurements [27]. The HABD is coloured red, with white arrows depicting the polarity of the HA-binding cleft. The extent of homodimer formation may also be regulated by the redox potential in the local environment. The splayed configuration of the homodimer derives from the small-angle X-ray scattering (SAXS) structure obtained with soluble LYVE-1 ectodomains (grey); the in vivo configuration remains uncertain. (**B**) HA-binding avidity is also regulated by clustering of LYVE-1 in the form of crosslinked macromolecular complexes with additional HA-binding partners (e.g., TSG-6, Versican and IαI heavy chain, represented by orange and violet boxes), and by disassembly of the sub-membrane actin meshwork that corrals the receptor and constrains LYVE-1 lateral mobility in the plasma membrane. The figure is adapted from ref. [32] with permission from the publisher.

Furthermore, like CD44, the consensus Link module of LYVE-1 was predicted to carry an extension, comprising a single N-terminal beta strand (β0) and three c-terminal beta strands (β7, 8 and 9), stabilised by a third disulphide bond. As discussed later in the review, these features have since been validated in the recently derived high-resolution crystal structures, with the exception that the region ascribed to the β8 and 9 strands is unstructured and instead forms part of the stalk-like membrane-proximal domain. Notably, this region also contains an unpaired cysteine residue that mediates LYVE-1 homodimerisation through formation of an intermolecular disulphide bridge, a feature not shared by CD44 and thus far unique within the Link superfamily [11,27,28,29]. Interestingly, homodimerisation places the HA-binding sites of the two monomer units in a mirror-image anti-parallel orientation (Figure 2), which implies either they each engage the same HA chain through the introduction of an intervening loop that reverses polarity, or each unit binds to a separate chain of opposite polarity. How this relates to HA binding in the context of an immune cell surface glycocalyx remains to be determined.

As regards HA binding in vitro, initial studies with recombinant soluble forms of the LYVE-1 extracellular domain expressed as Ig Fc fusion proteins established LYVE-1 as a *bona fide* HA receptor with little or no apparent affinity for the related glycosaminoglycans chondroitin or heparan sulphate and a minimal footprint of 12–22 sugar units, approximately twice as long as the minimal binding unit determined for CD44 [30]. Somewhat confusingly, however, when similar studies were carried out with either full-length LYVE-1 in transfected 293T fibroblasts or CHO cells, or with the native receptor in primary LECs, neither could be shown to bind HA to the extent observed with the soluble Fc fusion proteins. The enigma was eventually resolved when it was found that HA binding required the full-length receptor to be either crosslinked using divalent LYVE-1 antibodies or its level of expression raised above a critical threshold density [31]. Likewise, binding could be induced when the valency of HA itself was increased by prior crosslinking with its binding partner TSG-6, or densely coated on the surface of latex beads [31]. Together, these findings underline the avidity-dependent nature of the LYVE-1 • HA-binding interaction—the necessity for an individual HA chain to engage multiple receptors via a multiplicity of individual weak binding affinities in order to generate efficient binding (Figure 2 and ref. [14]).

This property, known as superselectivity [33,34]—a disproportionate increase in binding of a polyvalent ligand induced by a small change in receptor density, quantifiable in terms of a Superselectivity Index (SI), defined as α_R_—the slope of the binding curve on a double-logarithmic plot of ligand v receptor density, where the value of α_R_ is >1. Notably, LYVE-1 has a SI value of 3 [31], indicating that relatively minor changes in its surface density can act as an on/off switch for HA binding in vivo. Such superselective binding of HA may also be tuned by LYVE-1 homodimer formation in response to changes in the redox environment, given that dimerisation increases the binding affinity of the receptor by some 15× fold [27], and also by terminal sialylation of its O-linked glycan chains (see Figure 1 and ref. [35]), which is thought to limit LYVE-1 density in the plasma membrane through mutual charge repulsion.

## 3. Location of LYVE-1 Within the Lymphatic Network

**Figure 3:**
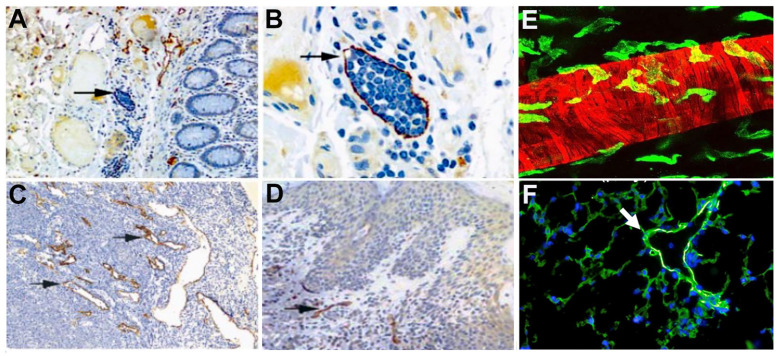
Representative expression of LYVE-1 in different tissue locations. Images are fixed sections of human colon (**A**,**B**), lymph node (**C**) and skin (**D**), highlighting lymphatic capillaries stained brown with LYVE-1 polyclonal Ab and immunopreoxidase conjugate, together with immunofluorescent-stained mouse skin (**E**) showing LYVE-1+ve macrophages (green) around an artery, and mouse lung (**F**) showing a single lymphatic vessel (white arrow, vivid green staining) surrounded by numerous LYVE-1+ve blood vessels (fainter green staining). Panels (**A**–**D**) are adapted from ref. [11] with permission from the publishers, and panel (**E**) is shown with permission from Vernique Angeli (University of Cote d’Azur) and Hwee Ying (National University of Singapore).

The identification of LYVE-1 as an HA receptor within the lymphatics emerged from immunohistochemical staining analyses of human and murine tissue that focussed initially on skin and intestine, firstly with polyclonal and later monoclonal antibodies [11,36]. These revealed the striking abundance of LYVE-1 in lymphatic capillaries and lymph node sinuses (Figure 3), a pattern distinct from CD44, which is widely expressed in many different cell types but whose expression within the vasculature is confined to blood rather than lymphatic vessels.

While subsequent studies have revealed that *Lyve1* can also be expressed by some blood vessels and tissue macrophages [37,38,39], as discussed below, this has not detracted from its widespread use as a lymphatic marker, most notably in studies on lymphangiogenesis in the developing embryo [40,41], in lymphoedema [42], and in nodal metastasis of tumours, both in animal models and human cancers (see, e.g., [43,44,45]).

**Figure 4:**
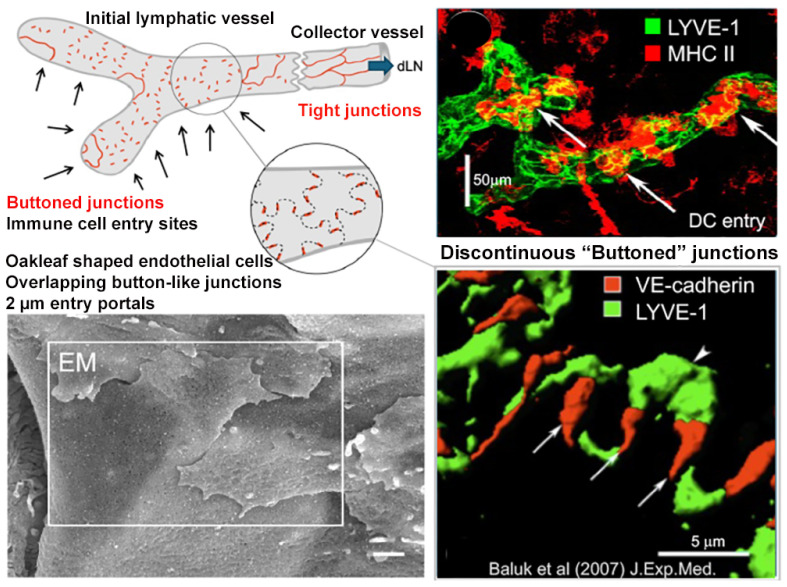
Localisation of LYVE-1 within the specialised inter-endothelial junctions of lymphatic capillaries. (**Top left** and **bottom right** panels): cartoon showing the key features of endothelial junctions within blind-ended lymphatic capillaries that serve as entry portals for immune cells (buttoned junctions) and high magnification confocal image of immunofluorescent antibody-stained section (trachea) showing location of LYVE-1 and VE-cadherin on overlapping flaps of the constituent oakleaf-shaped endothelial cells. (**Top right** panel): confocal image of MHC II +ve DCs adhering to LYVE-1-lined lymphatic capillaries prior to vessel entry in LPS-treated trachea. Arrows point to DCs inside the vessel lumen. (**Bottom left** panel): scanning EM image of the surface of a lymphatic capillary endothelial junction showing individual overlapping flaps. Arrows point to VE-Cadherin in buttons and arrowhead points to LYVE-1 between buttons. Images are adapted from [46] and reproduced with permission from the publishers.

In mammals, the lymphatic network typically starts in the periphery as blind-ended initial capillaries, which in turn lead to deeper, valved pre-collectors and collectors vested with contractile smooth muscle cells. Subsequently, these drain into serial lymph nodes and, finally, lymphatic ducts that empty back into the blood circulation. Within the initial capillaries, the constituent endothelial cells have a distinctive oakleaf shape, with scalloped borders (aka flaps) that interdigitate to form overlapping, “buttoned” junctions, distinct from the continuous, non-overlapping junctions of downstream collectors and blood vessels (Figure 4 and refs. [46,47]). So named because the overlapping flaps are buttoned at their sides by tight junction proteins including JAMs, ESAM and Claudins, and the adherens-junction protein VE-cadherin, these create portals of approximately 2–3 μm diameter that are sites of entry for fluids, macromolecules and immune cells [46]. It is at the tips of the lymphatic portals that LYVE-1 is concentrated [46], and from where it mediates the entry of immune cells such as DCs and monocyte/macrophages from the surrounding parenchyma, through chemokine-guided motility and adhesive interactions with HA in the incoming immune cell glycocalyx and LYVE-1 transmigratory cups as described more fully below [12,15,48,49,50,51]. Indeed, much remains to be understood about the workings of these LYVE-1 and CD31 lined portals, whose overlapping flaps were shown to undergo constant sliding and dynamic re-modelling in response to changes in interstitial fluid volume [52]. Moreover, it has been reported that adhesive contact between transiting DCs and lymphatic vessel endothelium triggers a rapid discharge of CCL21 from *trans* Golgi vesicles in the endothelium to its basolateral surface, in a co-ordinated mechanism for on-demand entry of appropriate immune cells to the lymphatic capillaries [53,54]. Buttoned junctions may also be sites for HA uptake into lymphatics; whereas smaller products of local HA turnover have hydrodynamic dimensions in the range of hundreds of nm that could enter by passive diffusion, larger HA molecules can in principle exceed 2 μm and hence may be taken up by the endothelium via LYVE-1-mediated endocytosis [55].

Besides lymphatics in peripheral tissues, LYVE-1 also decorates the draining lymph node sinuses, including the floor of the subcapsular sinus, transited by incoming-antigen-loaded DCs during immune surveillance and the cortical and medullary sinuses transited by naïve and antigen-activated lymphocytes exiting the nodes to rejoin the blood circulation [56,57]. In contrast to the initial lymphatics and nodal sinuses, LYVE-1 is typically absent from the partially zippered downstream pre-collectors and tightly zippered collectors, consistent with the role of these more impermeable, contractile vessels in the containment and pumping of lymph fluid rather than the entry of immune cells [46]. Nevertheless, as recently demonstrated in human skin [58], these pre-collector lymphatics can in fact express *Lyve1* and emerging evidence suggests the receptor may also be present within the peribronchial lymphatic collectors in mouse lung—seemingly unconventional vessels which lack smooth muscle investment and are believed to be hotspots for immune cell exit in pulmonary inflammation [59,60].

## 4. How and Where LYVE-1 Mediates Immune Cell Entry to Lymphatics

**Figure 5:**
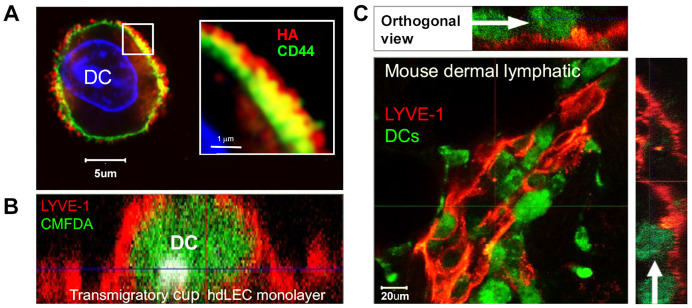
The HA glycocalyx and its role in DC entry to lymphatics via LYVE-1 transmigratory cups. (**A**) Confocal spinning disc image of mouse monocyte-derived DC showing details of the CD44 anchored HA glycocalyx (stained red using biotinylated versican VG1 domain reagent).A magnified area of the glycocalyx is detailed within the white bordered box. (**B**) Confocal image (z-section) of a chloromethyl fluorescin diacetate (CMFDA)-loaded DC adhering to the upper surface of a hdLEC monolayer showing formation of a LYVE-1 transmigratory cup in vitro. (**C**) Confocal images of DCs adhering to lymphatic capillaries in mouse skin via LYVE-1 transmigratory cups in vivo. White arrows point to DCs partially docked onto the cups. Direction of DC migration is through the endothelium (downwards in the upper image and towards the right in the right-hand image). Images are adapted from [15] (**A**) and [12] (**B**,**C**) with permission from the publishers.

The details of how LYVE-1 mediates immune cell entry and trafficking in lymphatics are still not fully clear, due in part to the technical difficulties in imaging such events in vivo and in real time. Nevertheless, recent studies have established that both dendritic cells and macrophages achieve such passage by means of adhesive interactions between LYVE-1 and a dense HA glycocalyx on their cell surface [31]. In DCs, this glycocalyx has been observed as a dense corona of some 200–300 nm thickness, synthesised primarily by the HA synthase HAS2 that generates long-chain high-molecular-weight polymers and anchored in the plasma membrane by CD44 (Figure 5 and refs. [12,16,61]). To date, no such HA glycocalyx has been detected in neutrophils, which instead are assumed to enter lymphatics by means of integrin rather than LYVE-1-dependent interactions [62].

Our current understanding of the mechanism by which LYVE-1 mediates the process of transmigration is based largely on confocal and videomicroscopic imaging of DCs interacting with primary LEC monolayers. An important caveat of such studies is that cultured monolayers fail to form the button junctions characteristic of lymphatic capillaries in intact tissues [63,64]. Nevertheless, due to their abundant expression of *Lyve1* they serve as a useful surrogate from which we have observed the process of transmigration to proceed through the following series of discrete steps. Firstly, in response to CCL21 released by the LECs, the DCs undergo polarisation to form a lamellipodium at the leading edge and an adhesive uropod at the trailing edge—the compartment within which the CD44-anchored HA glycocalyx becomes concentrated [15]. Secondly, following contact with the HA glycocalyx, LYVE-1 is redistributed into ring-like assemblages termed “transmigratory cups” on the LEC surface that envelop the DC [12] (Figure 5), analogous to the ICAM-1/VCAM-enriched cups that guide the transit of T-cells across blood vascular endothelium to the interstitium [65]. Both LYVE-1 transmigratory cup formation in lymphatic endothelium and CD44 redistribution in the DC uropod involve the submembranous actin cytoskeleton, which in each case restricts the lateral mobility of the two receptors. But whereas CD44 is directly tethered to the actin cytoskeleton through ERM (Ezrin, Radixin and Moesin)-binding motifs within its cytoplasmic tail [66], LYVE-1 lacks such motifs and is instead loosely corralled within submembranous actin fences [67]. It is not clear to what extent the clustering of LYVE-1 that occurs during cup formation is achieved by triggering of actin disassembly, or as a result of its engagement with an appropriately crosslinked configuration of HA in the DC glycocalyx. Thirdly, the DCs transmigrate, using transient adhesive interactions between LYVE-1 and the glycocalyx at the uropod and its actomyosin-mediated retraction to push and squeeze the cell body through the endothelium by chemokine-directed motion [15], likely at tri-cellular junctions [54]. Interestingly, engagement of LYVE-1 with pro-inflammatory HA fragments can also transduce signals via MAPK/ERK and src kinases, in addition to protein kinase-c, for the transient opening of buttoned junctions via VE-Cadherin phosphorylation and proteolytic degradation that would further facilitate immune cell entry [68,69,70].

**Figure 6:**
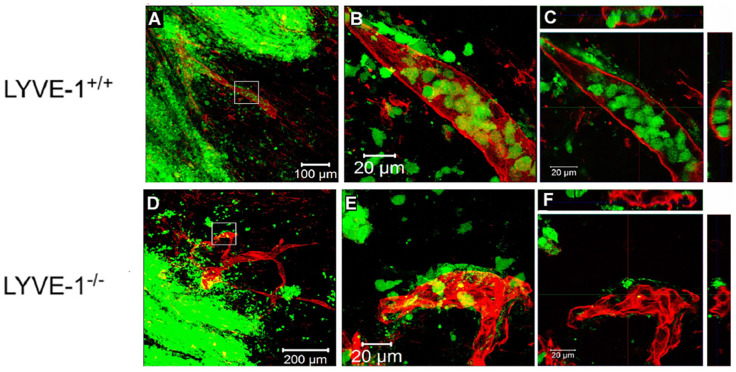
Impaired DC entry to mouse dermal lymphatics in *Lyve1^−/−^* mice. Confocal images (3D rendered z-stacks) of adoptively transferred (green) fluorescent DCs either within the lumen or logjammed around the periphery of lymphatic capillaries (podoplanin staining, red) in the ear skin of wild-type (**A**–**C**) and *Lyve1^−/−^* mice (**D**–**F**). The white boxed areas in (**A**) and (**D**) are further magnified in (**B**,**C**) and (**E**,**F**) respectively. (**C**) and (**F**) are orthogonal sections that depict DCs inside and outside individual lymphatic vessels Image is adapted from [12] with permission from the publisher.

In vivo confirmation of the critical role played by LYVE-1 • HA interactions during both lymphatic entry and trafficking of DCs has come from a comprehensive series of animal experiments with wild-type and *Lyve1* knockout mice, in which either endogenous or adoptively transferred DCs are mobilised to enter dermal capillaries and migrate to downstream LNs in response to the skin sensitising agent oxazolone. These studies established that either genetic deletion of *Lyve1* or pre-administration of LYVE-1 HA-blocking mAbs reduced DC migration to draining cervical nodes by as much as 60% through logjamming of the DCs around the outer surface of the lymphatic vessels (Figure 6 and ref. [12]). Note that under normal conditions, *Lyve1* knockout mice displayed no obvious phenotype, indicating the function of the receptor is particularly important in inflammation, when immune cells are mobilised to enter lymphatics. Similarly, ex vivo depletion of the DC • HA glycocalyx by enzymatic digestion with purified hyaluronidase prior to adoptive transfer and disruption of its re-assembly by co-administration of the HA synthase inhibitor 4-methylumbelliferone (4-MU, Hymecromone^®^), impaired lymphatic transit and migration to draining LNs by as much as 50% [15]. Furthermore, the importance of LYVE-1 • HA-mediated DC trafficking for immune activation was underlined by the demonstration that imposition of functional blockade by either LYVE-1 mAbs or *Lyve1* gene deletion impaired the development of T-cell responses to both dermally administered vaccine and DC-loaded antigen in skin-draining LNs [15]. In addition to LYVE-1 transmigratory cup formation, the rate and extent of DC entry to dermal lymphatics may also be controlled by changes in the surface density of the CD44 • HA glycocalyx, as evidenced from the results of experiments with the CD44 crosslinking mAb IRAWB14 that potentiates HA-binding; the greater the degree of crosslinking, the more the DCs adhere to the vessel wall but cannot transit to the vessel lumen [15]. Such changes in the organisation of the glycocalyx could be regulated in vivo, either through enhanced HA or CD44 biosynthesis or through formation of higher order HA complexes in the glycocalyx by interaction with known binding partners (e.g., TSG-6, Versican, IαI heavy chain), thereby tuning the rate of immune cell trafficking to the inflammatory status of the surrounding tissue environment [14,15,16,32]. It should also be noted that the levels of LYVE-1 in lymphatic endothelium are not static but subject to turnover, during inflammation (e.g., psoriasis, asthma), vessel maturation and neoplasia through uptake and degradation of the receptor via an early endosome/lysosomal pathway [55,71] and by a separate ectodomain shedding mechanism catalysed by the metalloproteinases ADAM-17 and MT1-MMP [72,73]. Curiously, LYVE-1 turnover is also controlled by the Circadian clock gene BMAL1 that regulates *Lyve1* diurnal expression in lymphatic vessels, curtailing immune cell trafficking to LNs during daylight hours when the receptor is downregulated and promoting it during sleeping hours when expression is reinitiated [74].

Besides DCs, confirmation that the LYVE-1 • HA axis is also important for monocyte/macrophage trafficking via lymph has been obtained from both in vitro and in vivo studies. In common with DCs, mature monocyte-derived macrophages were shown to synthesise and assemble a surface HA glycocalyx, as well as adhere and transmigrate inflamed LEC monolayers in vitro, in a LYVE-1 dependent manner [31,75]. Furthermore, in a mouse model of myocardial infarction (MI) induced by coronary artery ligation, where the resulting injured heart tissue is repaired and remodelled by Ly6C^low^ (lymphocyte antigen 6, C complex antigen) reparative M2-like macrophages and Ly6C^high^ inflammatory M1-like monocytes that promote lymphangiogenesis through endogenous VEGF-C release, both populations were shown to clear via the lymphatics to draining mediastinal LNs. Importantly, such clearance was markedly reduced in *Lyve1^−/−^* animals [75]. Moreover, monocyte clearance and injury repair post MI could be further augmented by exogenous administration of the lymphangiogenic growth factor VEGF-C, but this was apparent only in *Lyve-1^+/+^* and not *Lyve1^−/−^* animals [75]. Likewise, in a mouse model of sterile peritonitis elicited by Biogel^®^ bead injection, the exit of M1-like macrophages from the peritoneal cavity to draining mediastinal nodes was found to be significantly impaired in both *Lyve1^−/−^* mice and in wild-type mice administered with LYVE-1 HA-blocking mAbs (Bhattacharjee, S. and Jackson, D.G. (2026) [76]). Lastly, the LYVE-1 • HA axis has been shown to mediate the lymphatic adhesion and dissemination of certain extracellular microbial pathogens. Specifically, highly virulent strains of Strep pyogenes (Group A strep) bearing a dense HA capsule that cause tonsilitis, and in some cases rheumatoid arthritis and necrotising fasciitis in man, adhere specifically to both human and murine lymphatic endothelial cells via LYVE-1 [77]. Furthermore, in a mouse model of infection, such adherence was shown to enable lymphatic invasion and lymph node colonisation of these bacteria, as well as subsequent exit via efferent lymph to the blood circulation for systemic metastasis, thus identifying LYVE-1 as a critical host receptor during Group A strep pathogenesis [78,79].

## 5. LYVE-1 • HA Interactions Beyond the Lymphatics

Outside of lymphatics, *Lyve1* is also expressed by certain other cell populations in both mouse and human, including blood vessels in the lung and liver, and most notably, non-inflammatory M2-like macrophages, a yolk sac-derived tissue-resident population distinct from the more abundant pool of circulating monocytes and inflammatory M1-like macrophages. Unlike lymphatics, however, the precise function of the receptor in these locations is only partly understood at present.

In the case of macrophages, these were initially identified as scattered LYVE-1+ve/CD68+ve and F4/80+ve non-endothelial cells within human inflamed synovium and in developing mouse embryos, but have since been described in numerous other locations, from metastatic tumours to healing wound tissue [11,22,79]. Evidence of a functional link with lymphangiogenesis came initially from studies on mouse embryonic development (E14.5) in which LYVE-1+ve macrophages were observed to associate closely with newly proliferating lymphatics, particularly in the dermis, indicating a likely involvement in vessel sprouting [80]. More recently, these findings were confirmed in studies on the developing mouse heart from E12.5–16.5, which revealed LYVE-1+ve macrophages in intimate association with lymphatic capillaries undergoing growth and branching [37,81]. Further, in parallel experiments in which LYVE-1+ve hiPSC (human-induced Pluripotent Stem Cell) derived macrophages that mimic their tissue resident counterparts were co-incubated with LECs to induce vessel-like tube formation, the LYVE-1+ve cells were shown to associate and dissociate dynamically from the nascent vessels, guiding the extension of individual LECs and the assembly of new LEC • LEC junctions. Curiously, these interactions depended critically on the capacity of the LYVE-1+ve hiPSCs to form an HA glycocalyx [37], a function normally ascribed to CD44. Moreover, in early neonatal mouse hearts, it was reported that tissue-resident LYVE-1+ve, CCR2-ve macrophages can effect the repair of cardiac injury after MI without incurring the subsequent myocardial scarring seen in adults, by preventing the influx of M1-like inflammatory, pro-fibrotic CCR2+ve LYVE-1-ve monocytes from the circulation [82]. Again, the process was dependent on the formation of an HA glycocalyx by the LYVE-1+ve macrophages, which in this case functioned to protect the cells from apoptosis, as confirmed by macrophage-specific *Lyve1* deletion using a recently derived *CD68Cre ERT2 Lyve1^fl/fl^* mouse line [82]. Finally, LYVE-1+ve (MerTK, CD64, F4/80, CD11b, CD163 and CD206+ve) macrophages resident in the arterial wall in the mouse aorta (see also mouse skin, Figure 3 above) were shown to maintain vessel compliance through a LYVE-1-mediated interaction with HA on neighbouring intimal smooth muscle cells that was reported to regulate degradation of collagen by the metalloproteinase MMP-9 [38]. The interaction was shown to activate MMP-9 on the macrophages, as evidenced by the finding that inclusion of LYVE-1 HA-blocking mAbs abrogated the metalloproteinase activity and led to the accumulation of collagen with stiffening of the artery wall in the mouse aorta [38]. While a possible physical interaction between LYVE-1 and MMP-9 (in mice) has not yet been investigated, this cannot be ruled out in light of evidence that its closest relative CD44 co-localises with MMP-9 on the surface of human and murine tumour cells and can bind to the enzyme via its hemopexin (PEX) domain where it is thought to promote CD44 shedding and proteolytic activation of latent TFGβ to influence tumour cell migration and invasion [83,84].

Finally, studies in mice have reported that *Lyve1* is expressed on some blood vessel populations during early embryogenesis and some specialised vessels in the adult. Accordingly, the receptor is found within the yolk sac capillary plexus as well as venous and arterial endothelium throughout the mid-term mouse embryo, although these levels diminish progressively during later development [85]. Intriguingly, in adult mouse lung, LYVE-1 is hugely abundant not only in pulmonary arterioles and veins but also in bronchial epithelium, where its physiological roles remain completely unknown (Espinosa-Fematt, Royston, Baluk and Jackson, (2026) [86]). In addition, in both mouse and humans, LYVE-1 is particularly abundant in adult liver and spleen sinusoids [23,87,88,89], which have a porous, discontinuous endothelium specialised for macromolecule scavenging and immune regulation, somewhat analogous to lymphatic endothelium. Here, as in the lung, the role of LYVE-1 remains to be fully investigated, although it may well function as a scavenger for HA uptake and degradation—an important physiological process in which liver and spleen (together with lymph nodes) play central roles [17,18,19]. It is also possible that LYVE-1 acts as a docking receptor for pro-inflammatory HA complexes that are deposited on the surface of sinusoids in response to liver injury, specifically covalent adducts with the inter-alpha trypsin inhibitor heavy chain (SHAP) that promote extravasation of CD44+ve immune cells for the purpose of injury repair [5,90].

## 6. The Mechanics of the LYVE-1 • HA Interaction—An Unusual Sliding Mode of Adhesion and Detachment

**Figure 7:**
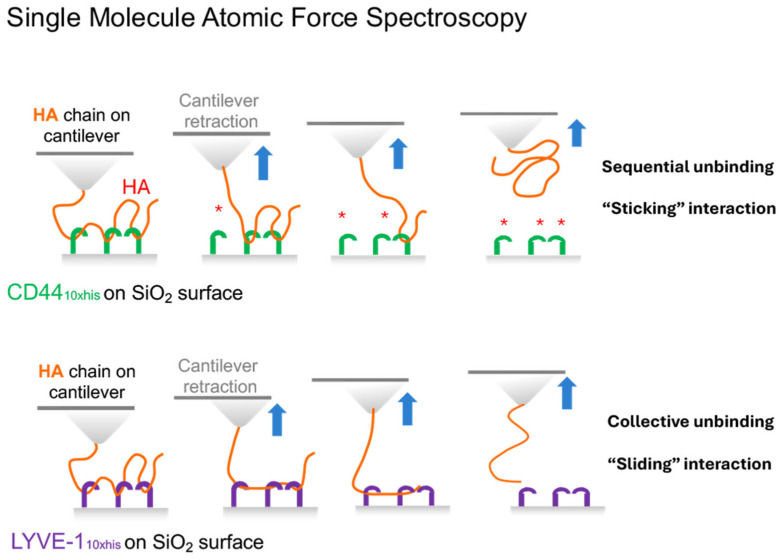
Distinctive HA-binding mechanics of LYVE-1 compared to CD44. Cartoon representation showing the Atomic Force Spectroscopy setup used to measure the binding and unbinding mechanics of HA and either CD44 (**top**) or LYVE-1 (**bottom**). A cantilever with single bHA (biotinylated HA) chains attached via their non-reducing ends was brought into contact with a SiO_2_ surface coated with either 10× histidine-tagged CD44 or LYVE-1 ectodomains, before progressive retraction (blue arrows) and measurement of the forces involved in the resulting bond breakages (depicted as asterisks). Unlike CD44, where the individual receptor/ligand bonds ruptured sequentially, signifying a conventional sticking interaction, those of LYVE-1 ruptured collectively, signifying an unconventional sliding interaction (see text). Image is modified and adapted from [26] with permission from the publisher.

Prior to solving the structure of the LYVE-1 hyaluronan-binding domain (HABD), the low binding affinity for HA and the marked dependence of the receptor on avidity [30,31] had already indicated that the nature of the HA interaction would be different to that of CD44. That this was indeed the case was first confirmed by probing the mechanics of HA binding/unbinding at the single molecule level using Atomic Force Spectroscopy (AFS)—a technique in which a cantilever bearing individual end-anchored HA chains is first brought into contact with a planar surface coated with c-terminally anchored receptor ectodomains before being slowly retracted, and the forces involved in rupturing the individual receptor • HA interactions measured (Figure 7).

Such AFS studies with CD44 have shown that the receptor behaves conventionally, with the individual bonds along each HA chain rupturing sequentially under forces of 30–60 pN [91]. Intriguingly, however, the behaviour of LYVE-1 is markedly different insofar as detachment from HA involves collective bond rupture and at lower forces (<30 pN) than for CD44, consistent with multiple receptor molecules acting together to resist the pulling force exerted on the sugar chain [26]. Accordingly, we have termed this unusual mode of engagement in LYVE-1 as a “sliding” interaction, to indicate attachment/detachment through movement of the HA chain along its contour, in contrast to the conventional sticking interaction of CD44 and other receptors in which attachment/detachment of the HA chain occurs without such movement. Further consistent with such an interpretation, comparative AFS measurements with looped HA [26] revealed a preferential (>100× fold faster) engagement of LYVE-1 with free (non-reducing) HA chain ends (zero force on rate *k*_on_ > 10^5^ M^−1^ s^−1^) as opposed to side-on engagement with internal HA sites (zero force on rate *k*_on_ < 10^2^ M^−1^ s^−1^). Taking into consideration that the (zero-force) off rate for LYVE-1 side-on binding to HA (*k*_off_ < 10^−2^ s^−1^) is far slower than that of CD44 (0.6 ± 0.1 s^−1^) [91], we concluded that the on-rate for side-on binding is at least two orders of magnitude slower than in CD44. In such a way, the unusual mechanics of the LYVE-1 • HA interaction likely enable LYVE-1 to capture the free termini of long HA chains, docking onto their non-reducing ends and threading them serially through adjacent receptors via a low-friction sliding interaction that supports rapid and reversible adhesion/de-adhesion.

## 7. Crystal Structure of the LYVE-1 HABD—How HA Sliding Is Achieved at the Atomic Level

**Figure 8:**
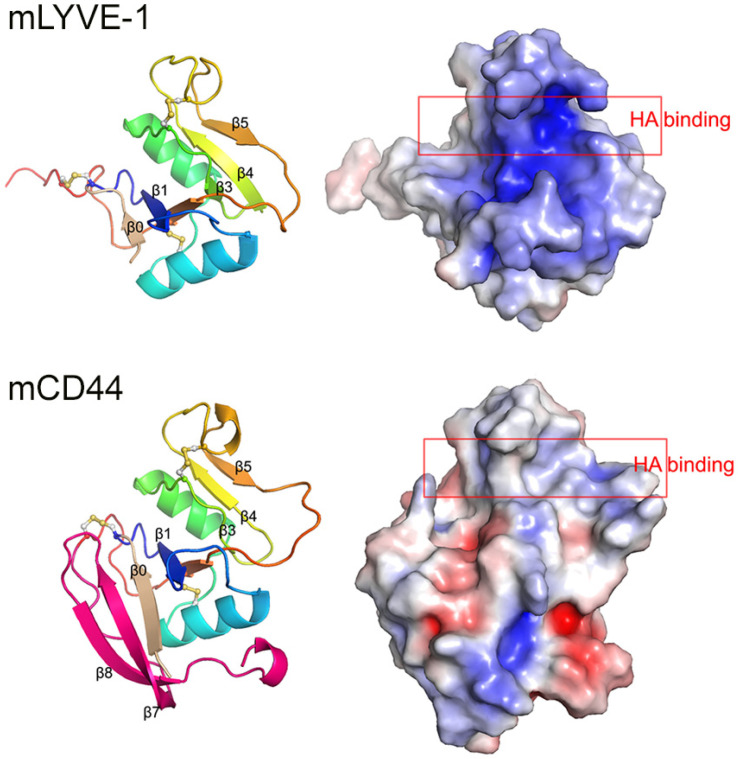
X-ray crystallographic structures of the HA-binding domains of mouse LYVE-1 and CD44 as ribbon diagrams at left (colouring depicts individual α and β strands) and space-filling representations at right showing surface electrostatic potential (coloured blue for positive charge and red for negative charge). The position of the HA-binding site in each case is boxed in red. Note the depth of the binding groove in LYVE-1 compared with CD44 and the prominence of the β4/β5 loop that forms an overarching clasp at its ceiling. Image is modified and adapted from [26] with permission from the publisher.

**Figure 9:**
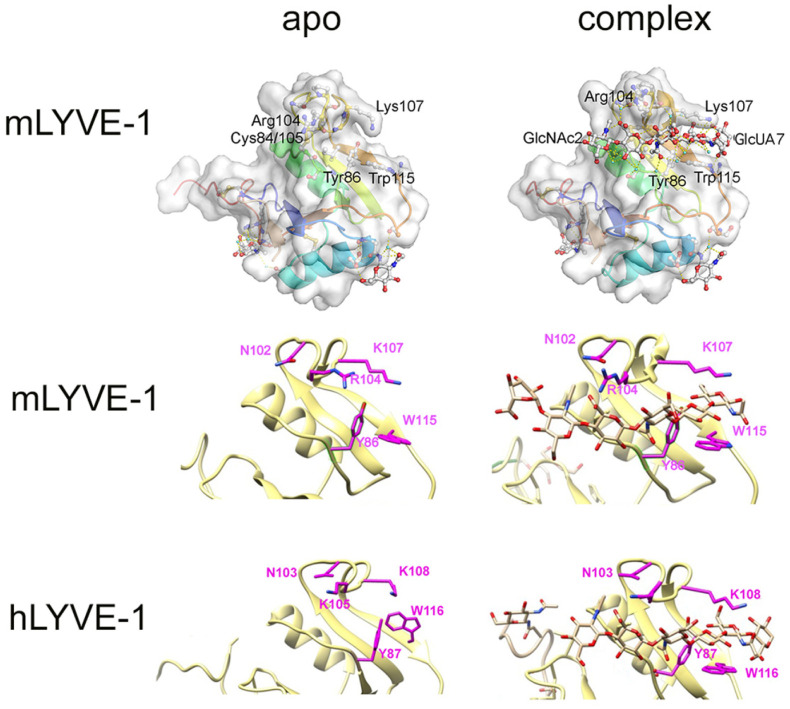
Key side-chain rearrangements in the LYVE-1 HA-binding cleft that enable the end-on entry and sliding mode of sugar interaction. Shown at the top are crystal structures of the unliganded (apo) mouse LYVE-1 HABD and HA8_mer_-bound complexes in semi-transparent space-filling format, illustrating the significant conformational changes that occur within the binding groove upon its engagement with HA. Note that neither GlcUA1 nor GlcNAc8 are resolved in the complex. Beneath are close-ups of the binding groove in mouse LYVE-1 detailing how the side chains of R104 and K107 in the roof swing upwards and outwards to accommodate HA entry (from the non-reducing end, right side in panel) and binding. Colouring depicts individual α and β strands. At the bottom are close-ups of the human LYVE-1 binding groove detailing how equivalent rearrangements in the side chains of K105 and K108 that accommodate HA-binding are accompanied by a twisting of the Y87 side chain and a downward rotation of the W116 side chain in the floor of the groove. Note that the latter side chains also obstruct side-on HA binding in the apo structure. Backbones are coloured yellow and featured side-chains are colouted pink. Images are modified and adapted from [26] with permission from the publisher.

The molecular basis for the end-on binding and sliding mode of HA interaction was deduced recently from the high-resolution crystal structures of unliganded mouse and human LYVE-1 HABDs and their HA-bound complexes (Figure 8 and Figure 9 and ref. [26]). In the first instance, these revealed that the HA-binding cleft in LYVE-1 occupies a deep groove, distinct from the shallower binding surface in CD44, and beneath a prominent loop between the β4 and β5 strands [92]. Notably, the binding groove and surrounding surfaces of the LYVE-1 HABD present a highly concentrated distribution of positive charge that contrasts with the more neutral binding surface in CD44 (Figure 8 and ref. [93]), indicating that engagement with HA occurs primarily through electrostatic interactions rather than the hydrophobic interactions which predominate in CD44 [26,92], as predicted earlier from the results of site-directed mutagenesis experiments [30].

Within the groove itself, the prominent β4/β5 loop at its upper surface forms an overarching clasp, lined by key basic residues (Lys107/Arg104 in mouse and Lys108/Lys105 in human), while the lower, opposing surface is lined by uncharged hydrophobic residues (Tyr86/Trp 115 in mouse and Tyr87/Trp116 in human), braced by the disulphide bond mCys84-Cys105/hCys85-Cys106 (Figure 9). Interestingly, the clasp-like loop is more flexible than its much less prominent counterpart in CD44 [92], and unlike CD44, it retains most of this compliance upon binding HA [26]. Importantly, however, access to the binding groove is partly obstructed by a number of key residue side chains, and these undergo conformational rearrangement upon HA binding, in a manner consistent with an end-on docking of the sugar chain, as deduced from the AFS analyses [26].

Accordingly, in mouse LYVE-1, the side chains of Arg104 and Lys107 in the clasp-like β4/β5 loop partly impede access to the distal end of the HABD and flip upward and sideways, respectively, upon HA engagement (Figure 9). This enables docking of the sugar via its non-reducing terminus and its directional advance through the binding groove. Moreover, in human LYVE-1, the side chain of an equivalent Lys108 that obstructs the binding groove moves sideways, while those of the critical Trp116 and hTyr87 at the floor of the groove rotate downwards to enable similar end-on HA docking and advancement (Figure 9). Animated videos depicting these various movements and the sliding of HA are viewable in reference [26]. The significance of such rearrangements for HA binding is further underscored by the fact that both hTyr87 and hTrp116 form part of the epitope for the hLYVE-1 HA-blocking mAb 3A [26].

Lastly, the structures of both the murine and human LYVE-1 HABDs reveal an unusual preponderance of bound water molecules (7–8, respectively) within and around the HA-binding groove—significantly more than the two structured waters in CD44. These participate in binding HA to the groove via H-bonding. Furthermore, evidence from molecular dynamics simulations suggests these undergo dynamic exchange, forming a ‘water cushion’ that likely lubricates the HA sliding interaction in LYVE-1 by means of rapid binding and unbinding of the sugar, a feature absent from CD44, where the direct interactions between HA and protein side chains mediate its conventional sticking interaction [26]. The extent of this water interface appears to be unique amongst HA receptors.

## 8. Likely Involvement of the Sliding Mode of LYVE-1 • HA Interactions in Lymphatic Entry and Downstream Migration

**Figure 10:**
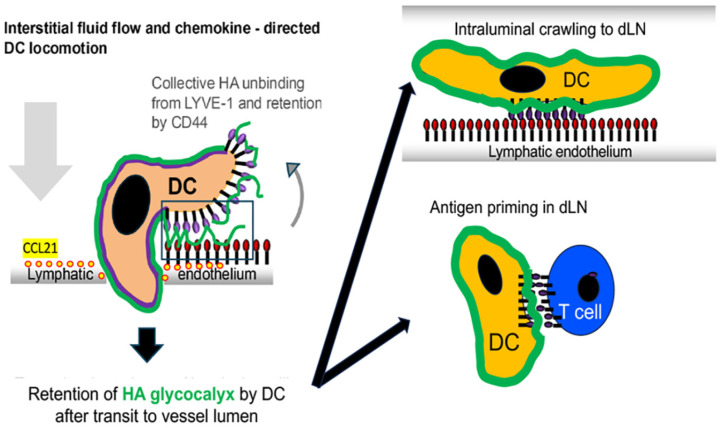
Hypothetical models depicting how the DC HA glycocalyx (green) and its sliding mode of engagement with LYVE-1 are thought to enable DC entry to lymphatics, subsequent crawling within the vessel lumen, and interaction with T cells in draining lymph nodes. Grey arrows indicate the direction of migration of the DC and the de-adhesion of its HA glycocalyx from CD44 on the endothelial surface. Black arrows point to the featured events following DC entry. Image is modified and adapted from [26] with permission from the publisher.

While distinct from the conventional “sticking” mode of adhesion exhibited by receptors such as CD44, the unconventional sliding mode of HA attachment and collective detachment displayed by LYVE-1 is well suited to its role in supporting the more transient, avidity-dependent adhesion that immune cells experience during lymphatic transmigration. Observed in vitro as a fluid-like locomotion when HA-coated dendritic cells adhere and migrate over a LEC monolayer [15], this equates in vivo to the amoeboid crawling and squeezing of HA-coated immune cells through the 2 μm button-like entry portals of lymphatic capillaries, under the low shear conditions within surrounding tissue interstices (<5 μN/cm^2^) and in response to chemokine-driven haptotaxis [46,48,49,53,94,95]. What remains to be determined is how the HA chains are configured on the surface of the immune cell and the extent to which their loops and free chain ends are exposed and available for engagement by LYVE-1 to initiate docking. Given the lower incidence but faster rate of LYVE-1 binding to HA free ends [26], we speculate the process begins with capture of these termini, followed by slower side-on binding to the more abundant HA loops—most likely in a co-operative fashion. After such engagement, transit to the vessel lumen would then proceed by peeling of the immune cell HA glycocalyx from endothelium through collective unbinding and reverse sliding on LYVE-1, accompanied by stochastic breaking and remaking of individual HA • CD44 bonds, under the tractive forces exerted by chemokine-directed DC locomotion (Figure 10). A further consequence of the rapid sliding mode of HA detachment from LYVE-1 is that it enables immune cells to retain rather than shed their HA glycocalyx following transendothelial transit, and in principle to maintain continuous adhesion to LYVE-1 on the luminal surface of lymphatic capillaries for intraluminal crawling towards downstream collectors and draining lymph nodes—a process previously observed for both DCs and T cells [96,97]. Within lymph nodes, retention of the HA glycocalyx is also predicted to be important for the transit of DCs across the LYVE-1-lined subcapsular sinuses and for the formation of an immune synapse via CD44 adhesion on T cells during antigen-specific immune activation [98].

## 9. Conclusions and Unanswered Questions

In conclusion, it would be misleading to suggest that LYVE-1 is the only receptor that mediates immune cell entry to lymphatics or that it acts alone in doing so. Much remains to be learned about the sequence of events downstream of LYVE-1 docking and how they regulate transendothelial transit. Does the process involve co-operation between LYVE-1 and those other adhesion receptors, including VE-cadherin, JAMs and CD99, which are known to mediate transmigration, and what are the dynamics of their individual contributions? Does LYVE-1 mediate immune cell entry to the same extent during both resting and inflammatory conditions when the classical adhesion receptors ICAM-1 and VCAM are upregulated? Is there crosstalk between the LYVE-1 • HA and integrin-mediated adhesion machinery under such conditions? Given the co-localisation of LYVE-1 and CD31 at the tips of the interdigitating endothelial ‘flaps’ within the button junctions of initial lymphatic capillaries, do the two receptors interact physically or co-operate to facilitate immune cell entry? Considering the degree to which HA binding is influenced by small changes in LYVE-1 surface density (i.e., superselectivity), it is tempting to speculate that active redistribution of the receptor (e.g., through localised depolymerisation of the submembranous actin cytoskeleton) could enhance immune cell motility during diapedesis. Could the sliding interactions between HA molecules and LYVE-1 within buttoned junctions act as a lubricant to enable the bellows-like expansion and contraction of lymphatic capillaries that occurs in response to changes in interstitial fluid pressure, as shown recently?

There is also much still to be learned about the function of LYVE-1 in its extra-lymphatic locations, such as the blood vasculature in the lungs—the organ that displays the highest levels of expression anywhere in the body. Does it mediate immune cell trafficking via both the pulmonary blood and lymphatic vasculature? Additionally, in M2-like tissue-resident macrophages, how does LYVE-1 interact with MMPs and how is this regulated by its interaction with HA, as has been reported for LYVE-1+ve macrophages in the walls of the mouse aorta? Does LYVE-1 substitute for CD44 in anchoring the HA glycocalyx in these macrophages, or does it act in concert with CD44 to do so? How does the sliding mode of LYVE-1 • HA interaction that enables immune cell transmigration apply to the functions of tissue-resident macrophages that interact with but do not necessarily exit via lymphatics? Does it allow for a more loosely tethered glycocalyx for interaction via LYVE-1 • HA • LYVE-1 sandwich formation? Lastly, could LYVE-1 • HA interactions be targeted for therapeutic benefit? Because they mediate a rate-limiting step in the generation of antigen-specific immunity, their blockade by LYVE-1 function-blocking mAbs could potentially impede the development of unwanted allogeneic responses, e.g., in tissue transplantation or, alternatively, autoimmune conditions. Indeed, in ongoing work, LYVE-1 • HA-blocking mAbs are already showing promise in preventing allograft rejection in a mouse model of corneal transplant rejection. The future design of newer, more effective blocking agents for use in human studies should certainly be assisted by the recent availability of high-resolution crystal structures for the mouse and human LYVE-1 • HA complexes.

## Data Availability

No new data were created or analysed in this study.

## Abbreviations

The following abbreviations are used in this manuscript:

AFS	Atomic Force Spectroscopy
DC	Dendritic Cell
ICAM-1	Intercellular Adhesion Molecule 1
LEC	Lymphatic Endothelial Cell
LV	Lymphatic Vessel
SAXS	Small Angle X-ray Scattering
VCAM-1	Vascular Cell Adhesion Molecule
